# Hyperscanning MEG for understanding mother–child cerebral interactions

**DOI:** 10.3389/fnhum.2014.00118

**Published:** 2014-03-04

**Authors:** Masayuki Hirata, Takashi Ikeda, Mitsuru Kikuchi, Tomoya Kimura, Hirotoshi Hiraishi, Yuko Yoshimura, Minoru Asada

**Affiliations:** ^1^Department of Neurosurgery, Osaka University Medical SchoolSuita, Japan; ^2^Department of Adaptive Machine Systems, Graduate School of Engineering, Osaka UniversitySuita, Japan; ^3^Research Center for Child Mental Development, Graduate School of Medical Science, Kanazawa UniversityKanazawa, Japan; ^4^Yokogawa Electric CorporationKanazawa, Japan

**Keywords:** hyperscanning, social interaction, mother–child interaction, MEG

## Abstract

Child development is seriously affected by social interactions with caregivers, which may lead to forming social minds in our daily life afterward. However, the underlying neural mechanism for such interactions has not yet been revealed. This article introduces a magnetoencephalographic (MEG) hyperscanning system to examine brain-to-brain interactions between a mother and her child. We used two whole-head MEG systems placed in the same magnetically-shielded room. One is a 160-channel gradiometer system for an adult and the other is a 151-channel gradiometer system for a child. We developed an audio-visual presentation system, which enabled a mother and her child to look at each other in real time. In each MEG system, a video camera was placed behind a half-mirror screen for visual presentation to obtain the subjects' facial expressions. The visual presentation system is capable of displaying not only real-time facial expression but also processed facial expression such as a still face or delayed facial expressions. A projector system displays the side-by-side face images of the mother and child, and the images are divided into each face using splitting mirrors and each face is displayed on the half-mirror screen in front of the other subject. To the best of our knowledge, our system is the first MEG hyperscanning system in a single shielded room, and may contribute to elucidating brain-to-brain interactions not only between a mother and her child but also in general inter-individual, brain-to-brain interactions.

## Introduction

The social interaction between a mother and her child has crucial roles for child development, which may lead to forming social minds in our daily life afterward. A few studies have investigated the mother–child interactions with respect to imitation behavior and gaze following using eye tracking or social contingency using a dual video system (Meltzoff and Moore, 1977; Nadel et al., 1999; Soussignan et al., 2006; Gredeback et al., 2010). However, those neural correlates are unclear. We hypothesized that the cognitive and emotional interactions between a mother and her child are induced by their behaviors and that such brain-to-brain interactions play a crucial role in forming social minds. A few papers have reported either only the mother's or the child's neural responses during mother–child interactions (Carver and Vaccaro, 2007; Musser et al., 2012). Numerous studies have employed hyperscanning methods while simultaneously recording brain activity from two or more adults during an interaction using electroencephalography (EEG), functional magnetic resonance imaging (fMRI), or near infrared spectroscopy (Montague et al., 2002; Babiloni et al., 2006; Dumas et al., 2010; Jiang et al., 2012; Sanger et al., 2012; Tanabe et al., 2012; Kawasaki et al., 2013). However, none of these reports have studied brain-to-brain interactions between a mother and her child. In addition, some of these methods, such as fMRI, present limitations such as low temporal resolution and difficulty in recording brain activity of awake children because of its small gantry space and noisy sounds.

Magnetoencephalography (MEG) provides high-resolution spatiotemporal dynamics of neuromagnetic fields during various cognitive and behavioral activities (Goto et al., 2011). Therefore, hyperscanning MEG may have a large potential to study brain-to-brain interactions. In addition, we accumulated the experience and know-how in recording neuromagnetic activities in awake children, despite the difficulty in acquiring stable MEG recordings for awake children because it is not easy to keep the children still (Kikuchi et al., 2011). In this study, we developed a hyperscanning MEG system to elucidate brain-to-brain interactions between a mother and her child or infant.

## Methods

**Figure 1:**
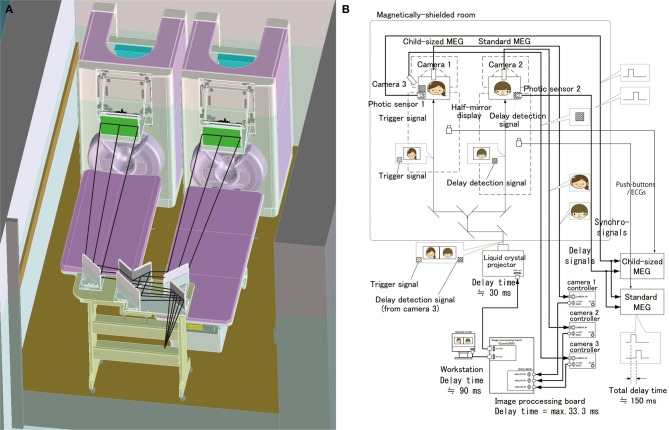
**Schematic and block diagrams of the hyperscanning MEG system. (A)** A standard MEG (right) and a child-sized MEG (left) were located in a single magnetically-shielded room. The optical image projecting system consisting of a liquid crystal projector, splitting mirrors and half mirrors displays a real time movie of each other's facial expression. **(B)** An audio-visual presentation and recording system was designed, particularly for simultaneous and synchronized recording. A visual presentation system was designed to record neuromagnetic cerebral interaction during face-to-face interactions between a mother and her child. This system has a real time visual image processor to present a real time movie of each other's facial expression.

Two MEG systems were housed in a magnetically-shielded room at Yokogawa Electric Corporation (Figure 1). One was a 160-channel whole-head gradiometer equipped with coaxial type gradiometers (MEG vision NEO, Yokogawa Electric Corporation, Kanazawa, Japan). This MEG system was used to record the mother's neuromagnetic activities. The other MEG system was a 151-channel whole-head gradiometer equipped with coaxial type gradiometers (PQ1151R, Yokogawa Electric Corporation, Kanazawa, Japan) that was designed to measure a child's or infant's neuromagnetic activities (Yoshimura et al., 2012; Kikuchi et al., 2013). The custom child-sized MEG system allowed measurement of the brain responses in children and infants, which would be difficult to obtain with conventional adult-sized MEG systems. In addition, the child MEG system ensures that the sensors are easily and effectively positioned within the range of the brain and that head movements are appropriately constrained (Johnson et al., 2010; Kikuchi et al., 2013). Both MEG systems are capable of simultaneously recording electroencephalograms (EEGs) with synchronized signals.

It is important to accurately measure children's brain activities because it is considerably difficult to keep children still in the gantry. We accumulated the experience and know-how to measure children's brain activities while minimizing their head motion and to analyze the measured data that may contain considerable motion artifacts (Kikuchi et al., 2011, 2013). In our setup MEG measurements are generally performed on two separate days. On both testing days, staff members make contact with the children and play with them along with the parent(s)/caretaker(s). We have a number of video programs with stories, particularly attractive to young children. We allow child subjects to select the video program according to their preferences. Before recording, we ensure that they are pleased with the selected video program. When performing MEG recording, one staff member (author Y.Y.) escorts the mother and her child into the shielded room, which is decorated with colorful pictures of Japanese cartoon characters and mimics an attractive vehicle adopted from an animation series popular with preschool children. During the measurements, the staff member stays in the shielded room, comforting and encouraging the child subject to maintain a steady body position when necessary. According to their evaluation, none of the participants endure high emotional tension or any other kind of discomfort during the measurements. We may alternatively present the selected movie and the other subject's facial expression (Supplementary Movie 1). This makes the child subject concentrate on the displayed visual stimuli and keep still. In addition, we designed a motion tracking system (RD60G-3D3SP/IRSO, Library, Co., Ltd., Tokyo, Japan) that allows the MEG systems to calibrate motion errors.

**Figure 2:**
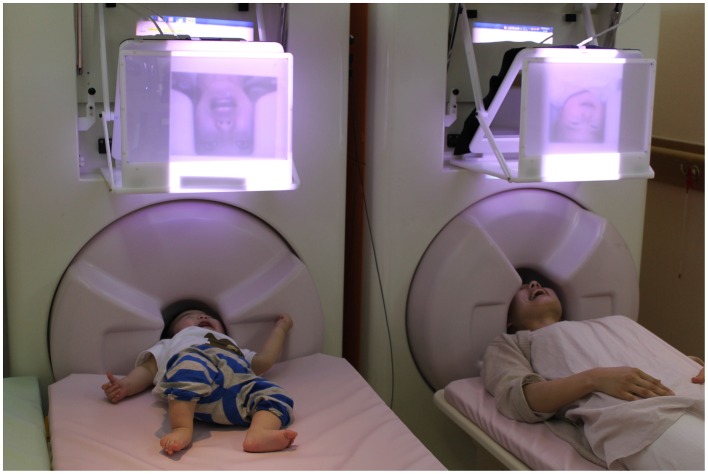
**MEG hyperscanning of a mother and her infant.** A mother and her infant look at the other's facial expression while neuromagnetic recording of their brain activities are performed. Note that the child keeps still while looking at his mother's face.

**Figure 3:**
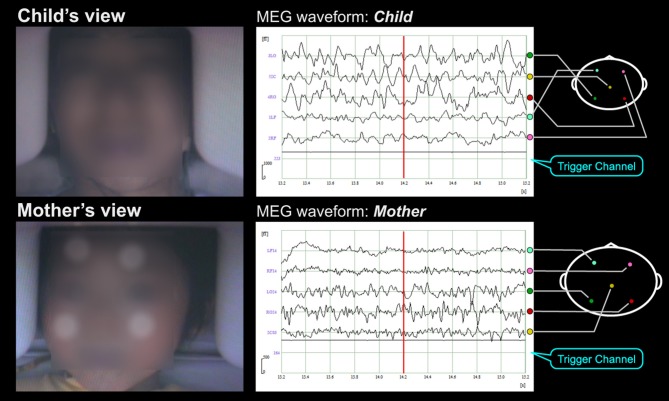
**Subjects' faces and MEG waveforms during MEG hyperscanning.** In this experimental paradigm, subjects were a mother and her child. Short movies and the facial expression of others were alternately displayed for 10 and 15 s, respectively, on the half-mirror screen in front of each subject (as indicated in the left pictures) while simultaneous neuromagnetic recording of their brain activities was performed (as indicated on the right waveforms). The lowest signals in the waveforms were the trigger signals not only to detect the change of visual stimuli but also to measure the time delay of facial expression movies. In the facial expression periods, the mother and her child looked at each other's facial expression. In the short movie periods, the mother and her child looked at the same short movies that were selected according to the child's interest. The subjects' faces were obscured in this supplementary movie to protect personal information. Four infrared circular markers (as indicated as white circular dots on the child's face on the movie) were attached on the child's face to correct the child's motion.

To measure the mother–child interaction in a face to face situation, we set up a real-time dual video presentation with facial expressions of the mother and child, in addition to a standard auditory recording and stimulation system (Figure 1). A half-mirror display was placed in front of each subject to present the other's face. Beyond the half-mirror display, a charge coupled device (CCD) camera (AS-808SP, MILS SYSTEMS Co., Ltd., Saitama, Japan) was placed to record the subject's facial expressions. Simultaneously-recorded mother's and child's faces were transferred to a workstation (Precision T5600, DELL Japan, Kawasaki, Japan) equipped with two image processing boards for input (DFG/MC4, ARGO CORPORATION, Suita, Japan) and output (Quadro2000, NVIDIA Corporation, CA, USA). In the workstation, an image acquisition software (ICImageController v3.2, ARGO CORPORATION, Suita, Japan) was used for image acquisition and a custom software compiled by Visual Basic (Visual Basic 8.0, Microsoft, USA) was used to combine both face movies into a single movie with the faces of the mother and child side by side. The combined movie is presented using a liquid crystal projector (IPSiO PJWX6170N, Ricoh Company Ltd., Tokyo, Japan) and is split into separated mother and child movies projected to two half-mirror displays using the specially designed splitting mirror system (Figure 1A). This video presentation system allows a mother and her child to see each other's facial expression in real time (Figures 2,3 and Supplementary Movie 1). The time delay for visual presentation was approximately 150 ms that includes a maximum of 33.3 ms for image acquisition by the image processing boards, approximately 90 ms for image processing by our custom software, and approximately 30 ms for image projection by the projector. In addition to this basic presentation, the system is capable of displaying processed facial expressions, such as a still face or facial expression with some time delay, which can be arbitrarily set using the custom software on the workstation. These movies of the subject's facial expression were also used as their behavioral data, which can be used to investigate the relationship between facial expression and neuromagnetic activities.

We ensure the synchronization of the two MEG systems by the following methods (Figure 1B). A trigger signal (a white square spot) is output by the projector at the onset of the change of each visual stimulus and is projected on the lower end position of the half mirror display. This trigger signal is detected by a photic sensor located at the corresponding position of the half-mirror display and the trigger timing is recorded into both MEG systems. In addition, facial expression movies are recorded aligned with MEG data by the trigger signals that are detected by the photic sensors from the half mirror displays and input into MEG data. Electrocardiograms and button responses are also monitored and aligned with the MEG data.

The time delay of the video display of facial expression is recorded trial-by-trial to correct it each time when we analyze MEG data and facial expressions. A trigger signal (a white square area) at the lower end is detected by the photic sensor 1 and is aligned with the MEG data. In addition, this trigger signal is recorded by camera 3 along with the subject's facial expression, which is captured into the image processing board and displayed on the half mirror in front of the other subject. This is also detected by the photic sensor 2 and aligned with the MEG data. The time difference between the two trigger inputs was the actual time delay of the present visual presentation (Figure 1B). We can correct this time delay when we analyze the MEG data by subtracting the time delay trial-by-trial.

Figure 3 and Supplementary Movie 1 show an example of our experimental paradigms. In this setup, subjects were a mother and her child. Short movies and the other's facial expression were alternately displayed for 10 and 15 s, respectively, on the half-mirror screen in front of each subject (as indicated in the left pictures) while simultaneous, neuromagnetic recording of their brain activities was performed (as indicated on the right waveforms). The lowest signals in the waveforms were the trigger signals not only to detect the change of visual stimuli but also to measure the time delay of facial expression movies. In the facial expression periods, the mother and her child looked at each other's facial expression. In the short movie periods, the mother and her child looked at the same short movies that were selected according to the child's interest. The subjects' faces were obscured in this supplementary movie to protect personal information. Four infrared circular markers (as indicated as white circular dots on the child's face on the movie) were attached on the child's face to correct the child's motion. As shown in Supplementary Movie 1, the child kept still while watching the short movie and even while looking at his mother's face.

The methods and procedures used in this study were approved by the Ethics Committee of Kanazawa University Hospital and were performed in accordance with the Declaration of Helsinki.

## Discussion

To the best of our knowledge, this is the first MEG hyperscanning system in a single room in which the participants are able to see each other's facial expressions in real time, although there have been reports of other hyperscanning recording systems using fMRI, near infrared spectroscopy, and electroencephalography (EEG) (Montague et al., 2002; Babiloni et al., 2006; Dumas et al., 2010; Jiang et al., 2012;). Only one study has reported a simultaneous, dual MEG recording system (Baess et al., 2012); however, each MEG system was placed at two distant institutions. We developed a simultaneous dual MEG recording system to study mother–child interactions using a real-time face presentation system.

fMRI studies have drawbacks while investigating temporal dynamics because hemodynamic changes have a delay of several seconds with respect to electrophysiological changes. In addition, it is difficult for children to keep still in a narrow and noisy MRI gantry. On the other hand, our system may reveal spatiotemporal dynamics of neural processes related to the mother–child interaction because MEG has high spatial and temporal resolution. Because our hyperscanning system is capable of simultaneously recording MEG and EEG, it is possible to detect radial magnetic components and deep brain signals, both features that are relatively difficult to detect using MEG alone.

Current research has only been able to study mother–child interactions with respect to imitation behavior, gaze following using eye tracking or social contingency using a dual video system (Meltzoff and Moore, 1977; Nadel et al., 1999; Soussignan et al., 2006; Gredeback et al., 2010). Only a few studies have used child MEG recordings because of the difficulty (Johnson et al., 2010; Bosseler et al., 2013; Kikuchi et al., 2013). In addition to attractive environments as described in the Method section, we established the broad region-of-interest (ROI) analyses to study the neural network (Kikuchi et al., 2011; Yoshimura et al., 2012; Kikuchi et al., 2013). We divided the child brain into 10 ROIs (five for each hemisphere). Although spatial resolution may fairly decrease, rather broad ROIs for MEG analyses allow tolerance to head motion, even if the motion is not negligible. A motion tracking and realignment system, which we newly plan to use in the present setup, may correct child motion if they are not too large to correct and may afford more precise ROI analyses.

The dual video processing system enabled us to manipulate from the workstation the visual stimuli using a custom software for image processing. The subjects' facial expressions are recorded by CDD cameras and transferred to a workstation by image processing boards. In the workstation, we can set an arbitrary time delay to more than 150 ms and display processed faces instead of real faces, e.g., upside-down, reversed faces or faces with modifications in some parts like eyes, mouth, or nose. Using this system, the still face paradigm (Brazelton et al., 1975) and dual video live-replay paradigm (Tremblay-Leveau and Nadel, 1995) can be used to investigate neural correlates of social contingency. However, the time delay of the dual video processing system is a potential limitation of the present setup. recent work indicates that subjective audiovisual simultaneity is achieved if the time difference is less than 150 ms (Hillock-Dunn and Wallace, 2012). This finding suggests that the time delay of our system is almost to the limit of the simultaneity but we can correct this time delay during the analysis of MEG data by subtracting the trial-by-trial time delay measured as described in the method section. In case the time delay is not acceptable, the 45-degree oriented half mirrors located in front of both subjects can display the each other's facial expression without video processing and time delay. In addition, in this case, we can record each subject's facial expression by positioning a camera at the back of the half mirror, aligned with MEG data, although we cannot use processed facial expressions. At present, we are introducing eye tracking systems into our dual MEG system, which provides another connection of brain activities with behaviors. Our hyperscanning MEG system is a promising tool to connect behaviors involved in the mother–child interaction with their neural correlates.

Regarding the analyses for mother–child cerebral interaction, we presently plan to use connectivity analyses methods, such as phase locking value analyses (Dumas et al., 2010, 2012) and partial directed coherence analyses, based on the Granger causality theory (Astolfi et al., 2010). Using these analyses, we may reveal brain-to-brain interactions between a mother and her child.

Our hyperscanning MEG system is not only applicable to mother–child cerebral interactions but also to adult–adult cerebral interactions. Our child MEG system works as well for adolescents and adults with a small head size. Social presence during mediated communication (Short et al., 1976; Biocca et al., 2003) may be a promising research topic using hyperscanning MEG because our system has a visual processing setup to present processed or artificial visual stimuli. Preliminary experiments suggest that face-to-face tasks using our real-time face presentation system results in restless and uneasy feelings in subjects. Actually, even psychologically-healthy subjects found it difficult to look at the eyes of another subject even for a few minutes. These restless feelings induced by face-to-face tasks are not a limitation but have a great potential for psychological research. We believe that this restless induction effect could be used in clinical examinations of psychological disorders. We are planning to use the hyperscanning MEG system to study different brain-to-brain interactions of psychological disorders such as autism spectral disorders and social phobia.

## Conclusion

We developed a hyperscanning MEG system in a single room that enables subjects to see each other's facial expressions in real time to investigate brain-to-brain interaction with high spatiotemporal resolution. Our dual MEG system is not only applicable to investigating the mother–child cerebral interactions but also to investigating adult–adult cerebral interactions.

## Conflict of interest statement

The authors declare that the research was conducted in the absence of any commercial or financial relationships that could be construed as a potential conflict of interest.

## Supplementary material

Supplementary Movie 1 **A movie showing MEG hyperscanning during an interactive task.** In this experimental paradigm, the subjects were a mother and her child. Short movies and another's facial expression were alternately displayed at least for 10 and 15 s, respectively, on the half-mirror screen in front of them (as indicated in the left movies) while simultaneous neuromagnetic recording of their brain activities are performed (as indicated on the right waveforms). The lowest signals in the waveforms were the trigger signals not only to detect the change of visual stimuli but also to measure the time delay of facial expression movies. In the facial expression periods, a mother and her child looked at each other's facial expression. In the short movie periods, a mother and her child looked at the same short movies that were selected according to the child's interest. The subject's faces were obscured in this supplementary movie to protect personal information. Four infrared circular markers (as indicated as white circular dots on the child's face on the movie) were attached on the child's face to correct the child's motion. Note that the child keeps still while looking at his mother's face.
